# Effects of mycelial spatial gradients on soil nematode community structure and ecological functioning under understory morel cultivation

**DOI:** 10.3389/fmicb.2026.1948707

**Published:** 2026-09-30

**Authors:** Bin Peng, Yingfei Xu, Wenxi Yang, Xun Li, Yan Zhang, Xiaolin Li

**Affiliations:** 1 Key Laboratory of Ecological Protection and Characteristic Industry Cultivation in Hengduan Mountain Area at Sichuan Minzu College of Sichuan Province Department of Education and Ganzi Prefecture, Kangding, China; 2 Sichuan Institute of Edible Fungi, Sichuan Academy of Agricultural Sciences, Chengdu, China

**Keywords:** energy flux, food-web structure, morel, mycelial spatial gradient, soil nematodes, understory cultivation

## Abstract

**Introduction:**

Understanding how spatially heterogeneous fungal growth reshapes belowground food webs is essential for evaluating the ecological consequences of understory mushroom cultivation.

**Methods:**

We investigated the spatial effects of understory cultivation of *Morchella sextelata* on soil nematode communities and food-web functioning in a pine forest. Soil samples were collected at 0 cm (R), 20 cm (R_20_) and 40 cm (R_40_) from the base of morel fruiting bodies, together with samples from an uncultivated control area (CK), and were analysed for nematode community composition, trophic structure, energy flux and environmental drivers.

**Results:**

A total of 603 nematodes belonging to 19 genera were recovered, with total abundance following the order R > CK > R_40_ > R_20_ (*p* < 0.05). *Aphelenchoides* and *Aphelenchus* were strongly enriched in R. PCA further revealed a clear separation of the R community from those of the other treatments (R^2^ = 0.58, *p* = 0.01). Fungivores and plant parasites were the dominant trophic groups, and their absolute abundances were significantly higher in R than in the other treatments (*p* < 0.05). The R community was overwhelmingly dominated by colonizer-persister-2 (c-p2) colonisers (76.09%), whereas the relative contributions of c-p3 and c-p4 taxa increased with distance from the fruiting bodies. Community organisation and food-web functioning also varied markedly across the spatial gradient. R_40_ exhibited the greatest evenness index, diversity index and structure index (*SI*), indicating a more highly structured and mature food web, whereas R showed the highest dominance and the lowest maturity index (*MI*), consistent with persistent resource-driven disturbance. Total nematode energy flux and the energy fluxes of individual trophic groups peaked in R, with bacterivores contributing the greatest biomass and energy flux. RDA and PLS further indicated that spatial distance from the mycelium-dense zone was associated with changes in soil carbon availability and enzyme activity, which in turn were linked to shifts in nematode community composition, trophic structure, energy flow and ecological functioning.

**Discussion:**

Our study reveals a spatially explicit pathway through which cultivated macrofungi reorganize soil faunal communities and energy flow, providing a multidimensional framework for assessing the ecological consequences of understory edible-mushroom cultivation.

## 1 Introduction

Morels (*Morchella* spp.) are rare edible and medicinal fungi belonging to the phylum Ascomycota. They are highly valued in international markets because of their distinctive flavour, high nutritional value and diverse health-promoting properties (Fu et al., 2025; Liu et al., 2025). However, wild morels have a short fruiting season, stringent environmental requirements and extremely limited yields, making natural production insufficient to meet growing market demand. Artificial cultivation has therefore become essential for the development of the morel industry and offers considerable economic value (Liu et al., 2025). *Morchella sextelata* is currently one of the most widely commercially cultivated morel species in China (Liu et al., 2022). It is highly popular in the market due to its attractive appearance, tender and meaty texture, and rich, savory flavour (Liu et al., 2023). Studies have shown that in the first year of cultivation, the mycelium of *M. sextelata* significantly overtook the native soil fungal community by reducing the α-diversity and niche breadth of the soil fungal community, resulting in a high yield of 12.39 ± 6.09 per plot (Zhang C. et al., 2023; Zhang Y. et al., 2023). However, continuous cropping is a major bottleneck for the sustainable cultivation of morels; for example, continuous cultivation of *M. sextelata* significantly reduces the pH and nutrient content of the mycorrhizal soil layer, while simultaneously increasing the abundance of pathogenic fungi, thereby reducing its yield (Liu et al., 2022). Therefore, a thorough understanding of the interaction mechanisms between M. sextelata and the soil is of great significance for stabilising yields and promoting the sustainable development of the industry.

In recent years, advances in cultivation technologies have driven a rapid expansion of morel production in China. Since commercial cultivation began in 2012, the cultivated area has increased from approximately 200 ha to 16,466 ha in 2021–2022 (Liu et al., 2023), while the total production of edible fungi in Sichuan Province has reached 2.4 × 10^6^ t (Liu et al., 2024). Against the broader policy background of ecological conservation and rural revitalization, understory economic activities have received increasing attention as a means of improving forest-resource utilisation and converting ecological assets into economic benefits (Chen et al., 2023). The cultivation of high-value edible fungi within forest ecosystems can enhance land-use efficiency while increasing both forestry productivity and farmers’ incomes (Liu et al., 2021). In particular, understory semi-natural cultivation of morels recreates key features of their natural habitat and takes advantage of forest shade, relatively stable temperature and moisture regimes, and abundant litter resources. By reducing the need for costly cultivation infrastructure, this production system has emerged as an important direction for morel cultivation (Xia et al., 2025). Previous studies have shown that understory mushroom cultivation can improve soil aeration, maintain soil structure (Kadiri and Mustapha, 2011), balance nutrient availability and enhance soil biological activity (Uzun, 2004). Fungal hyphal proliferation may also shape distinct bacterial community assemblages (Nazir et al., 2010).

Soil nematodes are among the most abundant and species-rich groups of metazoans in terrestrial ecosystems and occupy central positions within soil food webs. According to their feeding habits, they are commonly classified into bacterivores, fungivores, herbivores and predators–omnivores, each of which performs distinct ecological functions in organic-matter decomposition, nutrient mineralisation and energy transfer (Ferris et al., 2001). Because nematodes respond sensitively to environmental change, their community composition and diversity indices can provide integrated information on food-web complexity, disturbance history and ecosystem recovery (Xiao et al., 2026). Nematode-based food-web analysis has consequently become a powerful approach for resolving belowground ecological processes and patterns of energy flow (Gerhard et al., 2022). Ecological indices derived from the colonizer–persister continuum provide complementary information on different aspects of ecosystem organization. The maturity index (*MI*), enrichment index (*EI*), structure index (*SI*) and channel index (*CI*) respectively indicate food-web maturity, resource enrichment, structural complexity and the relative importance of bacterial- versus fungal-mediated decomposition pathways. Nematode metabolic footprints (*NMFs*) further quantify ecosystem functioning from an energetic perspective (Xiao et al., 2026). Together, these indices constitute an integrated framework for evaluating ecosystem structure and function and have been widely applied in agricultural management and ecological restoration (Chen et al., 2026).

Fungal communities can exert strong regulatory effects on soil nematode assemblages. For example, arbuscular mycorrhizal fungi (AMF) can increase the metabolic footprints of fungivorous and herbivorous nematodes through changes in root exudation (Yue et al., 2026). AMF hyphae also serve as food resources that support fungivore populations, whereas nematode feeding can, in turn, regulate AMF biomass and community composition (Jiang et al., 2020). Morel mycelia form dense trophic networks in soil and may therefore influence nematode communities through multiple pathways. They may directly provide high-quality food substrates for fungivorous nematodes, while their active decomposition of organic matter may indirectly alter the microhabitat selection of herbivorous and predatory nematodes by modifying carbon and nitrogen availability and soil pore structure. In addition, many soil fungi regulate nematode population densities by producing nematicidal secondary metabolites (Ayaz et al., 2024). Although morels are known to produce a wide range of secondary metabolites (Yang, 2020), whether these compounds affect nematode activity remains unknown. Incorporating morel mycelia into the conceptual framework of fungal–nematode trophic cascades could therefore both test the applicability of established interaction theories in edible-mushroom cultivation systems and reveal previously unrecognized mechanisms underlying morel–nematode interactions.

The cultivation, vegetative growth and fruiting of morels are closely associated with the surrounding soil environment. Using an enzyme-linked immunosorbent assay, Wang et al. (2019) quantified morel mycelial abundance in soil surrounding wild fruiting bodies. Mycelial density was highest immediately adjacent to the fruiting bodies and progressively declined with increasing distance, indicating that the fruiting-body vicinity represents a centre of mycelial activity. The extensive growth and spread of morel mycelia can directly alter soil physicochemical properties. For example, morel cultivation substantially increases soil organic-matter content (Zhang et al., 2020), while preferential mycelial uptake of nitrate nitrogen can induce species-specific changes in exchangeable calcium and other mineral elements (Fu et al., 2025). Mycelial development may also profoundly reshape the composition and organization of soil biotic communities. Morel cultivation has been shown to alter the diversity of bacterial and fungal communities as well as the stability of their co-occurrence networks (Fu et al., 2025; Liu et al., 2025; Zhang et al., 2026). Nevertheless, previous studies have focused primarily on changes in soil microbial communities, particularly bacteria and fungi (Zhang C. et al., 2023; Zhang Y. et al., 2023), or on soil enzyme activities (Yin et al., 2025). By contrast, little is known about how morel mycelial activity affects soil nematodes, despite their pivotal role in linking microorganisms with higher trophic levels in belowground food webs.

Importantly, morel mycelia are not distributed uniformly in soil but exhibit a pronounced spatial gradient centred on the fruiting body. This spatial heterogeneity in mycelial biomass may alter local resource availability, including organic-carbon inputs and nutrient availability, as well as soil enzyme activities, thereby generating distance-dependent effects on nematode communities. *In vitro* studies have suggested that morels possess an inherent capacity to resist grazing by fungivorous nematodes (Tayyrov et al., 2018) and can engage in complex interactions with microorganisms inhabiting the mycosphere (Zhang et al., 2026). However, under field conditions, it remains unclear how the spatial distribution of morel mycelia regulates the composition of nematode trophic groups, nematode ecological indices and energy-flow patterns within the soil food web. The environmental drivers and regulatory pathways underlying these effects have likewise not been resolved. This knowledge gap limits a comprehensive understanding of the ecological consequences of morel cultivation and constrains the development of scientifically informed and spatially refined management practices for integrated forest–fungus ecosystems.

Here, we used *M. sextelata* as the study organism and took advantage of the characteristic concentration of its mycelia around fruiting bodies. Soil samples were collected at distances of 0, 20 and 40 cm from individual fruiting bodies, together with samples from an uncultivated control area. Soil nematode communities were characterized and their diversity quantified through morphology-based identification. To clarify the spatial linkage between morel mycelial distribution and soil nematode responses, we formulated two research hypotheses: (1) The taxonomic composition, α-diversity, and trophic functional structure of soil nematode communities display distance-dependent spatial heterogeneity along the mycelial gradient of *M. sextelata*. The rhizosphere-like microhabitat surrounding morel fruiting bodies can drive the formation of unique nematode assemblages, which are significantly differentiated from those in distant bulk soil and uncultivated control soil. (2) The spatial variation in *M. sextelata* mycelial abundance profoundly modulates the functional traits of soil nematode communities. Key ecological indices, including the maturity index, channel index, and enrichment index, will undergo consistent gradient changes, which further restructure the complexity and stability of the soil food web and ultimately regulate the overall functional status of understory soil ecosystems. By resolving the effects of morel mycelia on nematode communities at a fine spatial scale, this study addresses an important gap in our understanding of belowground interactions in morel cultivation systems. The findings provide a scientific basis for evaluating and managing the long-term ecological consequences of morel cultivation and may contribute to the environmentally sustainable development of understory morel production.

## 2 Materials and methods

### 2.1 Study site

The study was conducted in Zhuangshang Village, Guzan Town, Kangding City, Sichuan Province, China (29°58′48″N, 102°12′36″E) (Figure 1). The region has a plateau-mountain climate characterised by distinct wet and dry seasons. The study site is located at an elevation of 1,850 m above sea level and has a mean annual temperature of 12.5 °C, a mean annual frost-free period of 210 days, mean annual precipitation of 850 mm and a mean annual relative humidity of 65%. The soil is predominantly dark-brown sandy loam. The overstorey is dominated by *Pinus densata*, whereas the shrub layer mainly comprises *Rhododendron lapponicum* and occasional *Viburnum acerifolium*, with sparse *goldenrod* plants also present.

**Figure 1:**
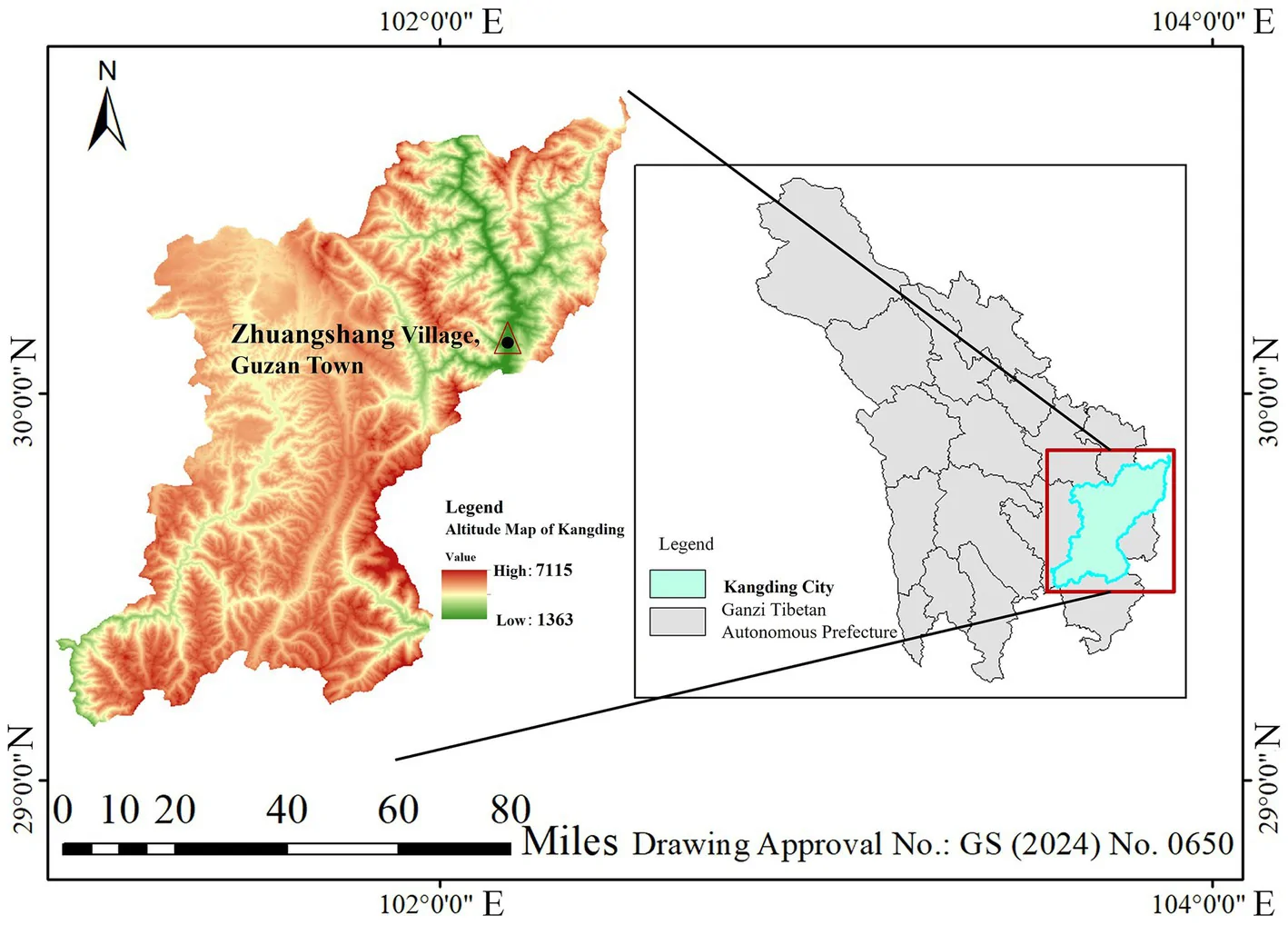
Geographic location and altitude of the study area.

### 2.2 Experimental design and soil sampling

In October 2024, a 10-m^2^ experimental plot was established beneath a *P. densata* stand in Zhuangshang Village. After the soil had been prepared, wheat-grain spawn of *M. sextelata*, produced in the laboratory, was evenly broadcast over the soil surface and covered with a layer of soil. During the morel cultivation period, the daily mean soil temperature at the 0–10 cm soil layer ranged from 0.7 °C to 16.3 °C (Supplementary Figure S1).

Soil samples were collected in April 2025 from a depth of 0–10 cm using a 4-cm diameter soil auger. All sampling tools were disinfected with 75% ethanol before use. Four sampling treatments were established according to the distance from the *M. sextelata* fruiting bodies: soil collected immediately adjacent to the fruiting body at 0 cm, representing the mycelium-dense zone (R); soil collected at distances of 20 cm (R_20_) and 40 cm (R_40_); and soil collected from an uncultivated control area (CK).

Topographic conditions and vegetation cover were consistent across all sampling sites. Each treatment included three independent replicates. For each replicate, five subsampling points were established within the designated sampling area: for the R group, five points were arranged around the base of the fruiting bodies; for the R_20_ and R_40_ groups, five points were distributed evenly along concentric circles at distances of 20 and 40 cm from the fruiting body, respectively; and for the CK group, five points were randomly selected within the uncultivated control area. After removing surface litter, soil was collected from each subsampling point, and the five subsamples were pooled in a sterile polyethylene bag and gently mixed to yield approximately 500 g of composite soil per sample. A total of 12 composite samples (4 treatments × 3 replicates) were obtained. Each composite sample was divided into three portions. The first portion was maintained under refrigerated conditions and rapidly transported to the laboratory. After stones, residual roots and other debris had been removed, the fresh soil was used for soil enzyme assays. The second fresh-soil portion was used for nematode community analysis. Following the same preliminary cleaning procedure, the samples were immediately transferred to centrifuge tubes for morphology-based identification. The third portion was used to determine soil physicochemical properties. These samples were air-dried, ground and sieved before the measurement of soil pH, electrical conductivity (EC), soil organic carbon (SOC), total nitrogen (TN) and total phosphorus (TP).

This study is based on a single-time snapshot sampling at the fruiting stage of *M. sextelata*. Therefore, our findings reflect soil nematode community characteristics under local environmental conditions at this specific phenological period and may not capture seasonal or inter-annual variations of soil nematode diversity.

### 2.3 Sample analyses

#### 2.3.1 Soil physicochemical properties and enzyme activities

Soil pH and EC were measured in soil–water suspensions prepared at ratios of 1:2.5 and 1:5, respectively, using a pH metre and a conductivity metre. Soil TN was determined using the Kjeldahl method, and SOC was quantified using an elemental carbon analyser (Wang and Anderson, 1998). Soil TP was measured by acid digestion followed by molybdenum–antimony colorimetry (Taylor, 2000).

The activities of β-1,4-glucosidase (BG) and cellobiohydrolase (CBH) were measured using fluorometric assays (Jackson et al., 2013). Urease activity (URE) was determined using the indophenol-blue colorimetric method, whereas catalase activity (CAT) was determined volumetrically (Xu et al., 2022).

#### 2.3.2 Extraction and identification of soil nematodes

Soil nematodes were extracted using the Baermann funnel method. For each treatment, three 50-g fresh-soil samples were separately placed in Baermann funnels, submerged in water and incubated for 48 h. This method effectively recovers free-living nematode communities, which are the primary focus of our functional community analysis because they serve as the most sensitive indicator species for soil food web dynamics and short-term ecological disturbances. The resulting nematode suspensions were collected and transferred to gridded Petri dishes for microscopic observation and enumeration (Cesarz et al., 2019). Nematodes were identified according to the Pictorial Keys to Soil Animals of China (Yin, 1998).

On the basis of feeding habits and morphological characteristics, the nematodes were assigned to four trophic groups: bacterivores (Ba), fungivores (Fu), plant parasites (Pp) and omnivores-predators (Op) (Bongers, 1990). According to their life-history strategies, nematode taxa were further assigned to five colonizer-persister classes, ranging from c-p1 to c-p5. This continuum represents a transition from r-selected strategies, characterised by short generation times, high reproductive capacity, rapid metabolism and strong tolerance to environmental stress, to K-selected strategies, characterised by long generation times, low reproductive capacity and high sensitivity to environmental disturbance (Bongers, 1990). The structure index and enrichment index were used to evaluate disturbance intensity, soil food-web dynamics and responses to resource enrichment (Ferris et al., 2001).

The mean fresh mass of an individual nematode belonging to each taxon, (W_t_), was obtained from the online Nematode Information System maintained by the University of California, Davis (Wan et al., 2022). Nematode energy flux (*F*), expressed as μg C 100 g^−1^ dry soil d^−1^, was calculated as the sum of energy allocated to production and respiration (Ferris, 2010):


$$F=\sum [N_{t}(0.1\cdot \frac{W_{t}/m_{t}}{12}+0.0159\cdot W_{t}^{0.75})]$$

where (*N_t_*), (*W_t_*) and (*m_t_*) denote the number of individuals, mean individual fresh mass and c-*p* value of taxon (t), respectively.

The allocation of carbon flux to the corresponding trophic compartments was subsequently calculated from the energy flux of each nematode group (Wan et al., 2022). A micro-food-web model was constructed comprising one basal-resource compartment, represented by detritus and living roots, and four nematode trophic nodes: Ba, Fu, Pp and Op. The feeding preferences of Op nematodes were fixed, whereas the proportional energy sources of the other trophic groups were determined according to the relative abundance of their available resources. Carbon flux through each trophic node, (*F_i_*), was calculated as follows:


$$F_{i}=(F+L)/e_{a}$$

where (*L*) represents the energy lost through consumption by higher trophic levels and (*e_a_*) represents the assimilation efficiency between trophic levels. Assimilation efficiencies were set at 0.60, 0.38, 0.25 and 0.50 for Ba, Fu, Pp and Op, respectively (Barnes et al., 2014).

### 2.4 Statistical analyses

Differences in nematode community characteristics, diversity index and related variables among treatments were evaluated using one-way analysis of variance in IBM SPSS Statistics 27. Duncan’s multiple-range test was used for *post hoc* comparisons, with statistical significance defined as (*p* < 0.05).

Origin 2024 was used to visualise nematode relative densities, trophic-group composition and the principal-component analysis of dominant nematode genera. Redundancy analysis was performed and visualised using Canoco 5. Partial least-squares structural equation models were constructed in R version 4.5.1 using the plspm, dplyr and pls packages to examine the relationships among soil physicochemical properties, nematode c-p classes, trophic groups, diversity indices and ecological functional indices.

General nematode diversity indices were calculated using PAST version 5, including the Shannon–Wiener diversity index (*H*), evenness index (*J*), dominance index (*λ*) and richness index (*D*). The ecological functional indices included the structure index (*SI*), enrichment index (*EI*), Wasilewska index (*WI*), maturity index of free-living nematodes (*MI*) and nematode channel ratio (*NCR*) (Ferris, 2010).

## 3 Results

### 3.1 Soil nematode community characteristics across the spatial gradient of understory morel cultivation

A total of 603 nematode individuals belonging to 19 genera were recovered. Total nematode abundance varied significantly among treatments and followed the order R (322 individuals) > CK (106 individuals) > R_40_ (97 individuals) > R_20_ (78 individuals) (*p* < 0.05; Figure 2A).

**Figure 2:**
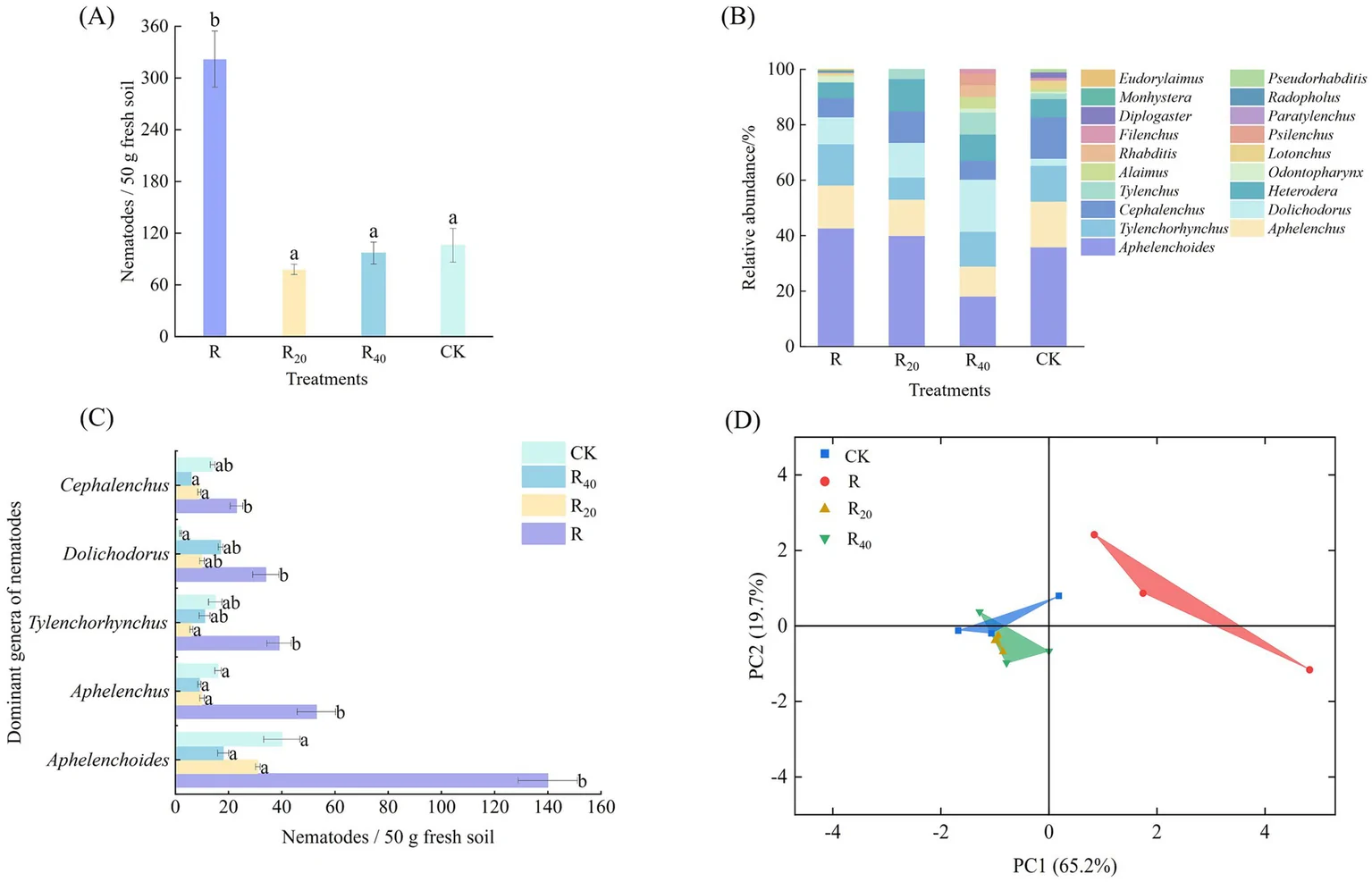
Characteristics of soil nematode community under different treatments. **(A)** Total number of nematodes in different treatments; **(B)** relative density of each genus; **(C)** number of nematodes in dominant genus; **(D)** principal component analysis. Different lowercase letters indicate significant differences among different treatments (*p* < 0.05).

In terms of relative abundance (Figure 2B), *Aphelenchoides* (18.14–42.69%), *Aphelenchus* (10.82–16.52%), *Tylenchorhynchus* (7.98–12.90%) and *Dolichodorus* (2.56–18.79%) were the dominant genera shared across all treatments. The relative abundances of *Aphelenchoides* and *Aphelenchus* were highest in R and lowest in R_40_. By contrast, *Dolichodorus* and *Tylenchorhynchus* were most abundant in R_40_ and CK, respectively, indicating a distance-dependent turnover in dominant genera. *Monhystera*, *Paratylenchus*, *Radopholus* and *Eudorylaimus* occurred exclusively in R. Because relative abundance does not necessarily represent population-level changes, absolute abundance was further analysed to clarify treatment effects (Figure 2C).

Analysis of absolute abundance further showed that the numbers of both *Aphelenchoides* and *Aphelenchus* were significantly higher in R than in the other treatments (*p* < 0.05; Figure 2C). Specifically, *Aphelenchoides* abundance in R reached approximately 140 individuals, more than four times that in R₄₀ (18), whereas *Aphelenchus* showed a similar pattern (53 in R vs. 35 in the other treatments). Although *Dolichodorus* had its highest relative abundance in R_40_, its absolute abundance remained highest in R (17 vs. 34 individuals per 50 g fresh soil).

Principal component analysis revealed clear differences in nematode community composition among treatments (Figure 2D). The first and second principal components explained 65.2 and 19.7% of the total variation, respectively. Samples from R were clearly separated from those from R_20_, R_40_ and CK along PC1, indicating a pronounced shift in community composition associated with the mycelium-dense zone (R^2^ = 0.58, *p* = 0.01). All samples were located within the 95% confidence ellipse.

Collectively, these results demonstrate that the effects of understory morel cultivation on soil nematode abundance and community composition were strongly distance-dependent. Areas immediately adjacent to the fruiting bodies were characterised by the enrichment of dominant genera and the localised occurrence of several otherwise undetected taxa.

### 3.2 Trophic and coloniser–persister structures of soil nematode communities across the spatial gradient

Fungivores (Fu; 28.96–58.21%) and plant parasites (Pp; 32.19–51.65%) constituted the predominant trophic groups across all treatments, whereas bacterivores (Ba) and omnivores–predators (Op) accounted for relatively small proportions of the communities (Figures 3A,C). No Op nematodes were detected in R_20_.

**Figure 3:**
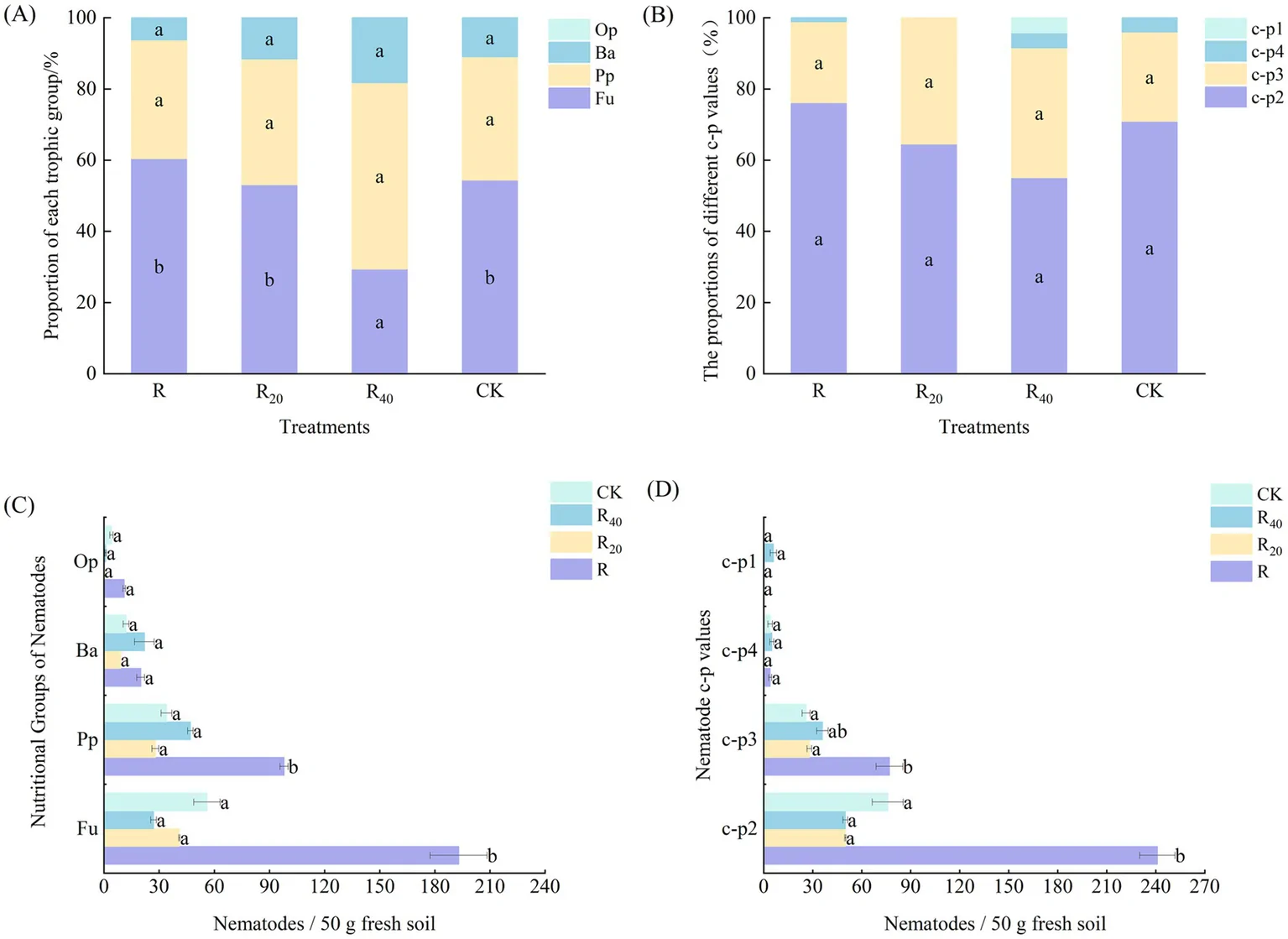
The proportion of each trophic group and c-p group of soil nematodes under different treatments. **(A)** Relative abundance of nematode trophic groups in different treatments; **(B)** relative abundance of nematode c-*p* value in different treatments; **(C)** number of nematode trophic groups in different treatments; **(D)** number of nematode c-*p* value in different treatments. Op is omnivores-predators nematode, Ba is bacterivores nematode, Pp is plant parasites nematode, Fu is fungivores nematode. Different lowercase letters indicate significant differences among different treatments (*p* < 0.05).

The relative abundance of Fu was highest in R and lowest in R_40_, whereas Pp exhibited the opposite pattern (*p* < 0.05; Figure 3A). In terms of absolute abundance, both Fu and Pp were significantly more abundant in R than in the other treatments (*p* < 0.05; Figure 3C), whereas no significant differences were detected among R_20_, R_40_ and CK. Although Pp had its highest relative abundance in R_40_, its absolute abundance remained lower than that in R because of the overall reduction in total nematode abundance at R_40_.

The nematode assemblages were dominated by c-p2 (55.04–76.09%) and c-p3 taxa (22.79–36.48%), whereas c-p1 and c-p4 taxa accounted for only minor proportions of the communities; no c-p4 nematodes were detected in R_20_ (Figures 3B,D). The relative abundance of c-p2 taxa was highest in R and lowest in R_40_. Relative to CK, the proportion of c-p3 taxa increased by 10.43 and 11.39% in R_20_ and R_40_, respectively, but decreased by 2.30% in R. The absolute abundances of both c-p2 and c-p3 taxa were significantly higher in R than in the other treatments (*p* < 0.05), with the enrichment of c-p2 taxa being particularly pronounced. No significant differences in their absolute abundances were observed among R_20_, R_40_ and CK.

These findings indicate that understory was associated with increased nematode abundance within the mycelium-dense zone, driven primarily by the pronounced proliferation of coloniser-type c-p2 taxa.

### 3.3 Soil nematode diversity and ecological indices across the spatial gradient

As shown in Table 1, nematode evenness, represented by the evenness index (*J*), was significantly highest in R_40_, followed by R_20_ and CK, and was lowest in R. The dominance index (*λ*) exhibited the opposite pattern: it was significantly highest in R, intermediate in R_20_ and CK, and lowest in R_40_. Neither the Shannon–Wiener diversity index (*H*) nor the richness index (*D*) differed significantly among treatments. Nevertheless, both indices reached their highest values in R_40_, whereas their lowest values were observed in CK and R_20_, respectively.

**Table 1:** Diversity indices of soil nematodes under different treatments.

Treatments	Diversity index			
	*H*	*J*	*λ*	*D*
R	1.66 ± 0.03a	0.61 ± 0.06a	0.27 ± 0.03b	1.65 ± 0.14a
R_20_	1.74 ± 0.07a	0.90 ± 0.03b	0.21 ± 0.03b	1.64 ± 0.07a
R_40_	2.09 ± 0.16a	0.96 ± 0.02b	0.13 ± 0.02a	2.22 ± 0.34a
CK	1.57 ± 0.38a	0.84 ± 0.08b	0.20 ± 0.05b	1.94 ± 0.33a

The number of replicates, *n* = 3; different lowercase letters within the same column indicate statistical significance at the 0.05 level.

The evenness indices of R_20_ and R_40_ approached 1, indicating highly even distributions of nematode taxa. Thus, the community in R was strongly dominated by a small number of abundant taxa and consequently exhibited the highest dominance. By contrast, R_40_ supported the greatest evenness, diversity and richness, particularly with respect to evenness, together with the lowest dominance, indicating a more evenly distributed and comparatively stable nematode assemblage.

Relative to CK, both R_20_ and R_40_ had higher structure index (*SI*) and enrichment index (*EI*) values, with the highest values observed in R_40_ (*p* < 0.05). Neither the Wasilewska index (*WI*) nor the nematode channel ratio (*NCR*) differed significantly among treatments. *WI* followed the order R > R_20_ > CK > R_40_, whereas *NCR* followed the order R_40_ > R_20_ > CK > R.

The maturity index of free-living nematodes (*MI*) did not differ significantly between R and CK but showed a slight increase with increasing distance from the fruiting bodies, following the order R < CK < R_20_ < R_40_. Both *SI* and *EI* reached their maximum values in R_40,_ with the increase in *SI* being particularly pronounced, indicating that the soil food web at this distance was more highly structured. Notably, *MI* was not highest immediately adjacent to the fruiting bodies and was instead slightly lower in R than in CK, suggesting that food-web maturity was not enhanced within the mycelium-dense zone. In contrast, R_40_ was characterised by the highest *SI* and evenness and the lowest dominance, collectively indicating a more developed food-web structure and comparatively high community stability.

### 3.4 Energy flux and biomass of soil nematode trophic groups across the spatial gradient

Understory morel cultivation significantly affected energy fluxes, represented by metabolic footprints, across soil nematode trophic groups (Figure 4). Relative to CK, total nematode energy flux increased in all treatments except R_20_. The largest increase occurred in R, where both total energy flux and the energy fluxes of all trophic groups reached their maximum values (Table 2).

**Figure 4:**
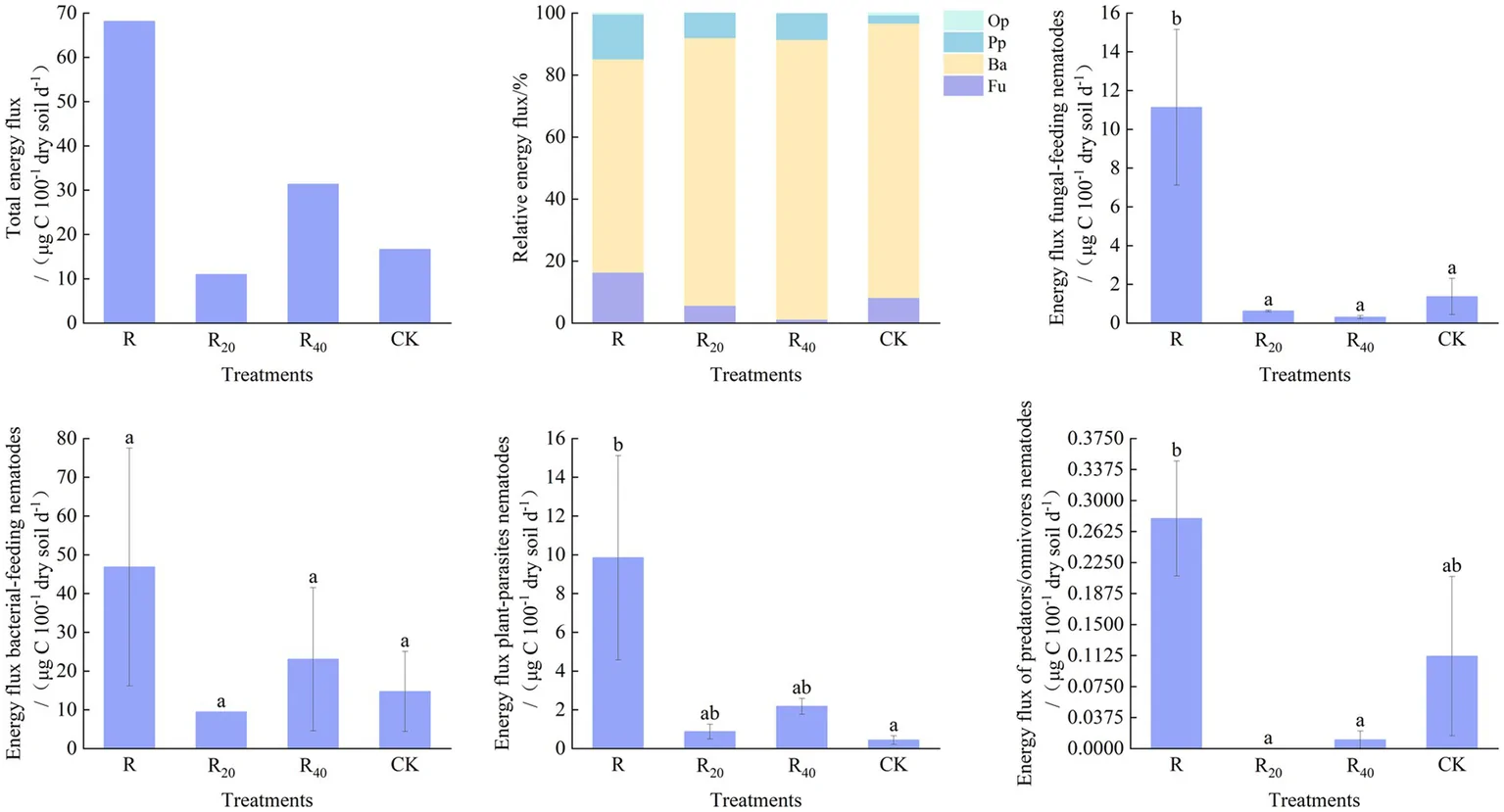
Effects of different treatments on nematode energy flux and relative energy flux. Different lowercase letters indicate significant differences among different treatments (*p* < 0.05).

**Table 2:** Ecological function indices of soil nematodes under different treatments.

Treatments	Ecological function index				
	*SI*	*EI*	*WI*	*NCR*	*MI*
R	28.82 ± 2.19a	49.89 ± 0.11a	2.15 ± 0.46a	0.09 ± 0.02a	2.25 ± 0.04a
R_20_	40.15 ± 4.26a	50.00 ± 0.00a	1.93 ± 0.36a	0.18 ± 0.00a	2.36 ± 0.04a
R_40_	58.35 ± 4.16b	53.57 ± 3.57a	1.02 ± 0.34a	0.33 ± 0.15a	2.38 ± 0.05a
CK	35.08 ± 7.48a	49.38 ± 0.62a	1.92 ± 0.27a	0.17 ± 0.05a	2.33 ± 0.11a

The number of replicates, *n* = 3; different lowercase letters within the same column indicate statistical significance at the 0.05 level.

Among the trophic groups, bacterivores contributed the greatest energy flux, followed by fungivores and plant parasites, whereas omnivores–predators made the smallest contribution. The energy fluxes of fungivores, plant parasites and omnivores–predators were significantly higher in R than in CK (*p* < 0.05). These results indicate a pronounced spatial concentration of nematode energy metabolism within the mycelium-dense zone. The microenvironment immediately surrounding morel fruiting bodies substantially enhanced energy flow through the basal soil food web, particularly through the bacterivore-mediated pathway.

Bacterivore biomass was also dominant across all treatments, followed by the biomass of plant parasites and fungivores, whereas omnivore–predator biomass was consistently lowest (Figure 5). The biomass of every trophic group was significantly greater in R than in the other treatments, and R exhibited the largest increase relative to CK.

**Figure 5:**
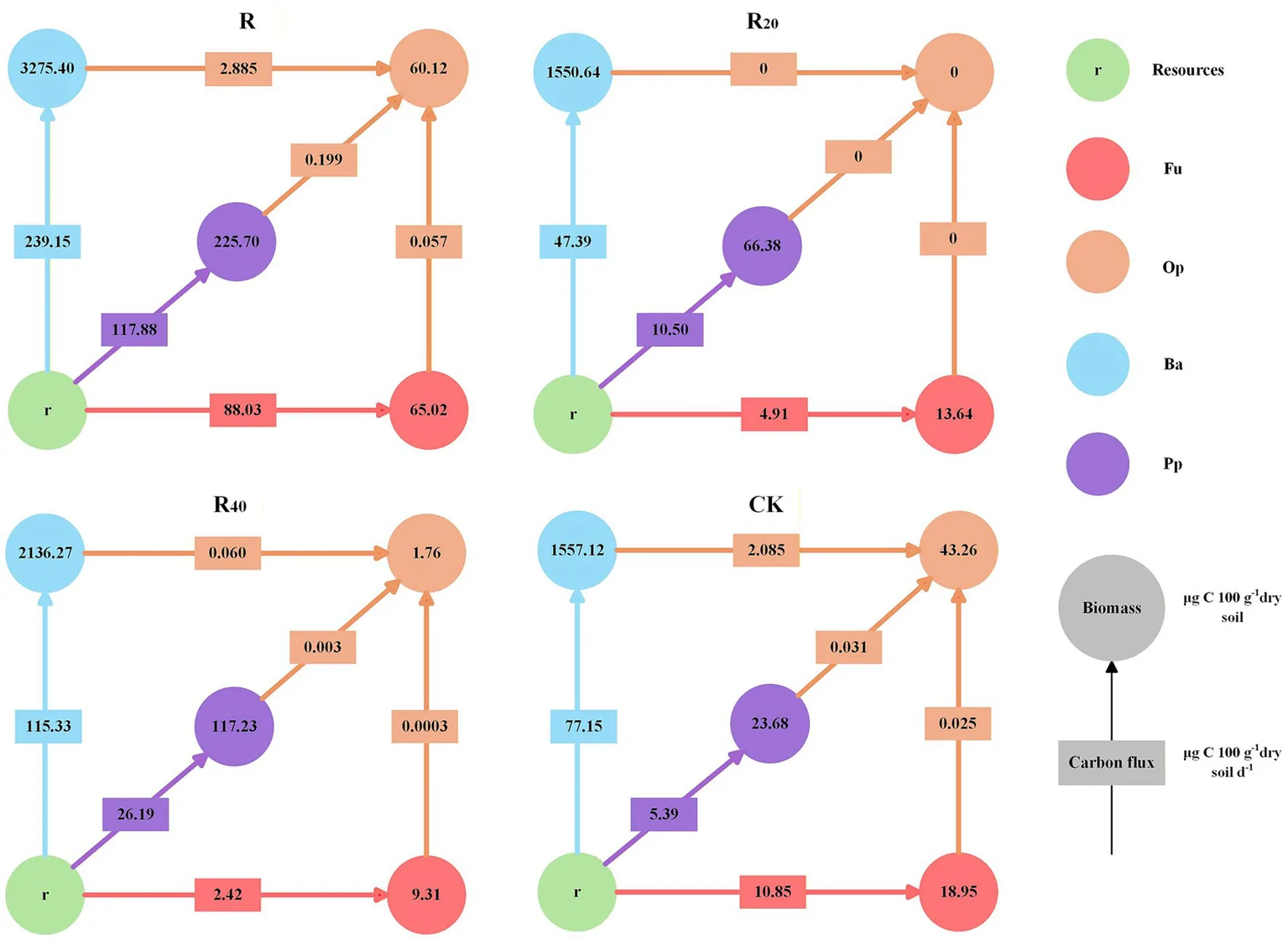
Effects of different treatments on energy structure of nematodes. Numbers in circles represent biomass and values on arrows represent carbon flux (μg C 100 g^−1^ dry soil d^−1^).

Across the spatial gradient, the biomasses of fungivores and omnivores–predators tended to be lower in R_20_ and R_40_ than in CK. By contrast, both groups increased markedly in R. These findings show that the mycelium-dense zone adjacent to morel fruiting bodies strongly promoted the accumulation of total nematode biomass. This spatial effect was particularly pronounced for bacterivores and plant parasites, highlighting the role of the morel mycosphere in shaping the distribution of biomass within the soil food web.

### 3.5 Redundancy analysis of soil properties and nematode community attributes

Redundancy analysis was used to evaluate the associations between soil physicochemical properties and nematode community attributes (Figure 6; Supplementary Table S1).

**Figure 6:**
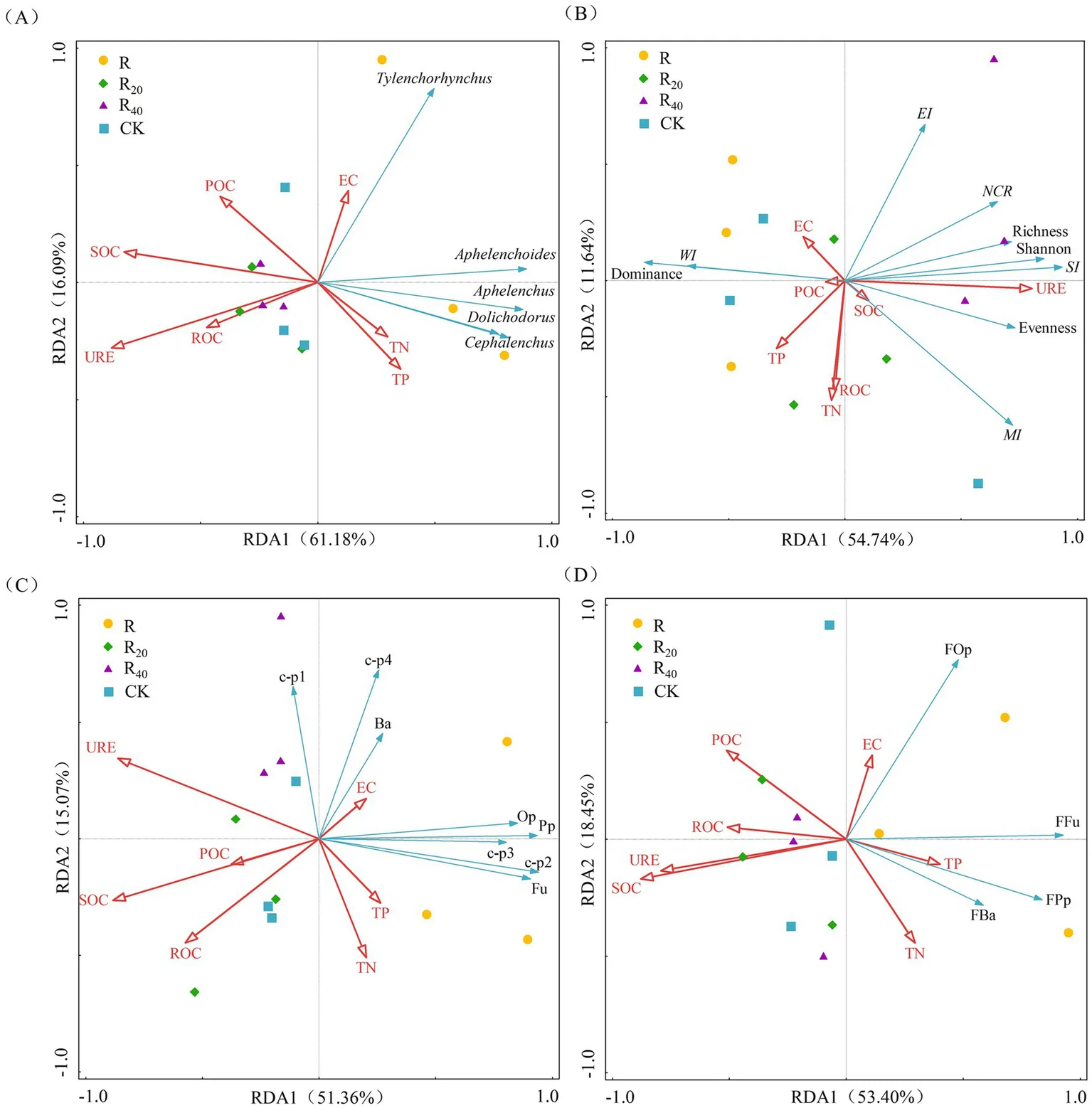
RDA analysis **(A)** soil physical and chemical properties and dominant genera; **(B)** soil physical and chemical properties and index; **(C)** soil physical and chemical properties and c-*p* value and nutrient groups; **(D)** soil physical and chemical properties and energy flux.

For dominant nematode genera, the first two constrained axes jointly explained 77.27% of the total variation (Figure 6A). Urease activity (URE) made the largest contribution, explaining 49.1% of the variation (*p* = 0.008), followed by particulate organic carbon (POC), which explained 13.5% (*p* = 0.032).

For nematode diversity and ecological indices, the first two axes explained 66.36% of the total variation (Figure 6B). URE was again the primary explanatory variable, accounting for 36.6% of the variation (*p* = 0.006), followed by SOC, which accounted for 17.0%, although its contribution was only marginally significant (*p* = 0.06).

For trophic groups and c-p classes, the first two axes explained 66.43% of the total variation (Figure 6C). SOC was the strongest explanatory factor, accounting for 41.3% of the variation (*p* = 0.002), followed by URE, which explained 15.7% (*p* = 0.006).

For nematode energy fluxes, the first two axes jointly explained 71.85% of the total variation (Figure 6D). SOC made the largest contribution, explaining 41.9% of the variation (*p* = 0.002). The remaining environmental variables made relatively small and statistically non-significant contributions.

Taken together, these results identify URE and SOC as the principal predictors of nematode community composition, trophic structure and energy flux across the spatial gradient of understory morel cultivation.

### 3.6 Partial least-squares structural equation modelling of soil properties and nematode community attributes

A partial least-squares structural equation model was constructed on the basis of the RDA results. The model showed a satisfactory overall goodness of fit (GoF = 0.6968) and relatively high explanatory power for the endogenous variables (Figure 7).

**Figure 7:**
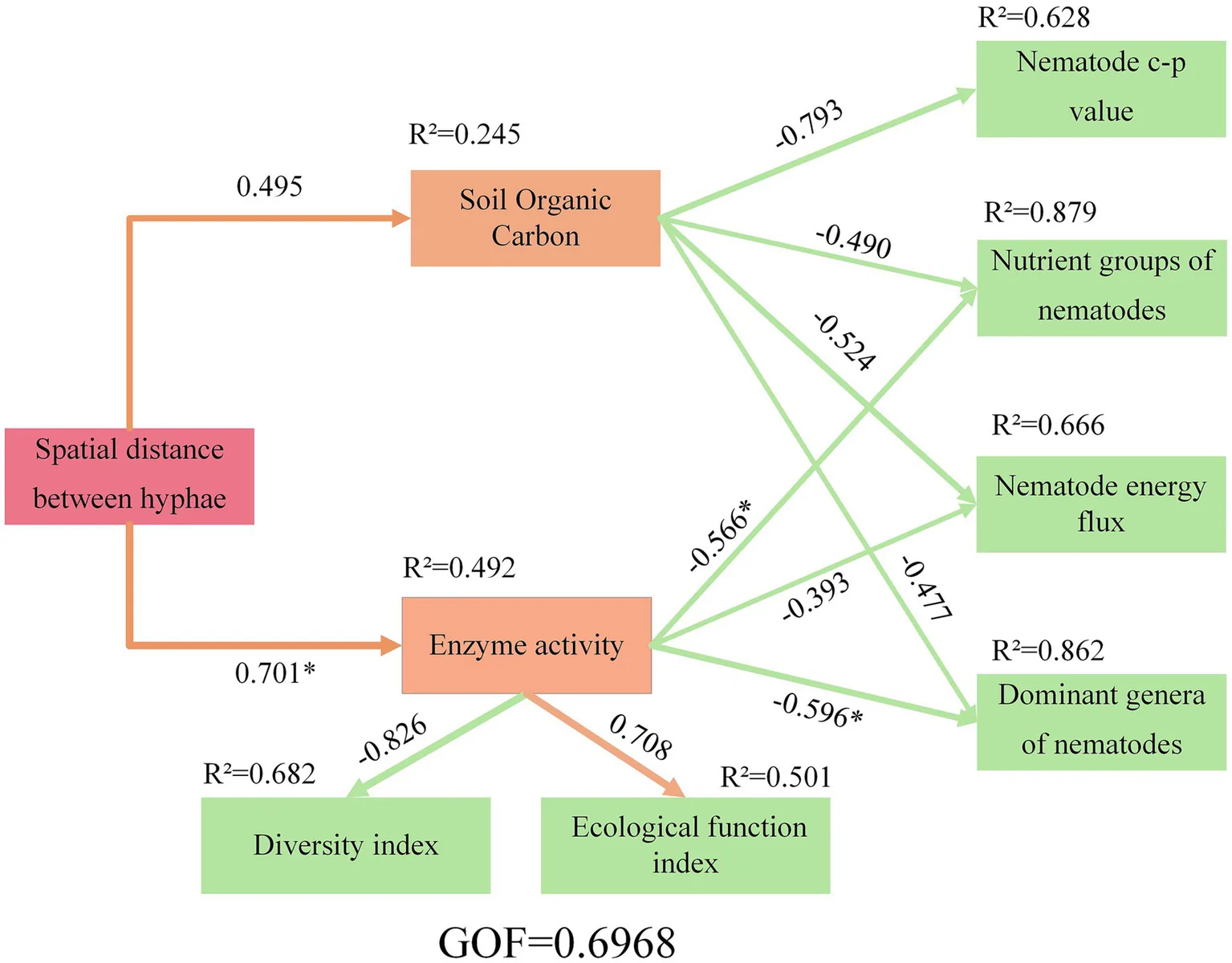
Structural equation model of soil physical and chemical properties of soil nematode. The *R*^2^ values in the figures represent the proportion of variance, and GOF indicates the goodness of fit. *indicate that the path coefficient is significant at the *p* < 0.05 level.

The spatial-distance gradient exerted a significant positive effect on soil enzyme activity, with a path coefficient of 0.701. Through its positive effects on SOC and enzyme activity, the spatial gradient further exerted negative effects on nematode trophic-group composition, nematode energy flux and dominant nematode genera. Soil enzyme activity was negatively associated with nematode diversity indices but positively associated with ecological functional indices, with path coefficients of −0.216 and 0.708, respectively.

The model therefore revealed a pathway through which spatial variation in morel mycelial influence altered soil chemical and biochemical properties and subsequently drove changes in nematode community composition, trophic structure, energy flow and ecological functioning within the understory soil food web.

## 4 Discussion

### 4.1 Spatial structuring of soil nematode communities under understory morel cultivation

Our results demonstrate that understory morel cultivation markedly increased total nematode abundance in the mycelium-influenced soil and substantially restructured nematode community composition. Nematode abundance was strongly concentrated in the mycelium-dense zone (R) and declined sharply at greater distances from the fruiting bodies, although the response was not strictly monotonic between R_20_ and R_40_. Several genera, including *Aphelenchoides*, *Aphelenchus* and *Monhystera*, were either preferentially enriched in or restricted to R, revealing pronounced spatial heterogeneity consistent with previous observations (Peng et al., 2026). Fungivores constituted a major trophic group and reached their highest relative abundance in R, identifying the immediate vicinity of the fruiting bodies as a resource hotspot for fungivorous nematodes. This enrichment may primarily reflect the direct provision of food substrates by abundant fungal mycelia and mycelial residues (Ruess et al., 2000). In addition, fungal exudates, including organic acids, amino acids and soluble carbohydrates, may stimulate microbial activity and thereby indirectly promote nematode proliferation (Hemmerling et al., 2022). The absolute-abundance data further supported this interpretation, as significantly more fungivorous nematodes were recovered from R than from the other treatments (*p* < 0.05). By contrast, the higher relative proportion of plant parasitic nematodes in R_40_ largely reflected the sharp decline in fungivore abundance and the concomitant reduction in total nematode abundance rather than an absolute increase in plant parasites.

The RDA results (Figure 6; Supplementary Table S1) identified urease activity (URE) and soil organic carbon (SOC) as major environmental predictors of nematode community structure and trophic composition, with the dominant genera generally showing negative associations with these variables. URE activity was lowest in R (Supplementary Table S2), potentially reflecting accelerated substrate turnover and changes in the local physicochemical environment associated with intensive mycelial metabolism. Previous work has shown that declining soil pH can directly constrain the expression and catalytic efficiency of extracellular enzymes (Sinsabaugh et al., 2008). The PLS-SEM analysis further indicated that spatial distance from the fruiting body was positively associated with SOC and enzyme activity, with path coefficients of 0.495 and 0.701, respectively. In turn, SOC and enzyme activity were negatively associated with both nematode community structure (path coefficients of −0.447 and −0.490, respectively) and trophic-group composition (−0.596 and −0.566, respectively; *p* < 0.05).

These negative relationships may arise from resource depletion and microenvironmental modification driven by intense fungal metabolism during fruiting-body development. On the one hand, rapid utilisation of labile carbon substrates by the mycelium, together with the release of organic acids, may accelerate substrate turnover and ultimately constrain URE activity through changes in substrate availability. This interpretation is consistent with the ecological stoichiometric framework proposed by Sinsabaugh, in which microbial extracellular-enzyme production is regulated by microbial nutrient demand and resource limitation (Sinsabaugh et al., 2009). On the other hand, soil pH was lowest in R (Supplementary Table S2), and acidic conditions have been reported to suppress the transcription, secretion and catalytic performance of some fungal extracellular enzymes (Snyman et al., 2019), providing a plausible explanation for the reduced URE activity observed in this zone. Such mycelium-induced acidification may also act as an environmental filter on nematode communities. The enrichment of *Aphelenchoides* and *Aphelenchus*, which have been reported to tolerate relatively acidic environments (Liu et al., 2019), together with the previously observed enrichment of acidophilic Acidobacteria in R (Zhang et al., 2026), supports the possibility that local acidification contributes to community filtering around morel fruiting bodies. This interpretation is consistent with the view of Yeates that nematode community composition provides a sensitive integrative indicator of changes in soil properties such as pH and organic-matter availability (Yeates, 2003).

R also supported the highest abundances of *Dolichodorus* and *Tylenchorhynchus*, while *Monhystera*, *Paratylenchus*, *Radopholus* and *Eudorylaimus* were detected exclusively in this zone. Together, these genera span several trophic positions, including bacterivores, plant parasites and omnivores–predators, suggesting that the area surrounding morel fruiting bodies functions as a spatially concentrated resource hotspot capable of supporting multiple trophic pathways. Fungal mycelia may directly support fungivores and exert chemotactic effects on fungal-feeding nematodes (Kitagami and Matsuda, 2022), whereas plant-derived rhizodeposits can provide favourable feeding environments for ectoparasitic plant nematodes (Zhang et al., 2011). At the same time, carbon-supported bacterial proliferation may increase food availability for bacterivores (Yeates et al., 1993). The accumulation of nematodes at lower trophic levels, including bacterivores and plant parasites, can in turn provide prey resources for higher-trophic-level taxa such as *Eudorylaimus* (Khan and Kim, 2006). These concurrent responses indicate convergent exploitation of a locally enriched carbon-input hotspot across multiple trophic levels. Ferris and Matute showed that patterns of co-occurrence among nematode trophic groups can reflect the structural complexity and energy-transfer pathways of soil food webs (Ferris and Matute, 2003). The coexistence of multiple trophic groups in R is therefore consistent with locally intensified trophic interactions, even though the broader indices of food-web maturity were highest farther from the fruiting bodies.

The dense nematode aggregation in R may also provide favourable conditions for nematophagous fungi and other antagonistic microorganisms. These microbes can actively capture and infect nematodes through specialised trapping structures and extracellular enzymes (Li et al., 2015; Yang et al., 2007), and such parasitic activity is often density-dependent, with infection rates increasing as nematode populations become more concentrated (Jaffee et al., 1993). Although nematophagous microbes were not directly quantified in this study, the high nematode abundance in R likely provides favourable conditions for their proliferation, representing a potential top-down regulatory mechanism warranting further investigation.

Analysis of the coloniser–persister continuum provided further evidence for strong spatial filtering. The R community was overwhelmingly dominated by c-p2 taxa, which accounted for 76.09% of all nematodes, whereas the proportions of higher-c-p taxa were comparatively low. With increasing distance from the fruiting bodies, the contribution of c-p2 taxa declined and c-p3 and c-p4 taxa became more prominent, particularly in R_40_, with CK generally occupying an intermediate position. Low-c-p colonisers typically dominate disturbed or resource-enriched environments, whereas higher-c-p persisters are more characteristic of stable and structurally mature soils (Bongers, 1990). Bongers and Korthals further reported that nutrient enrichment can selectively favour opportunistic nematodes, thereby reducing maturity indices (Bongers et al., 1997). Consistent with this ecological framework, the absolute abundance of c-p2 taxa was significantly greater in R than in all other treatments (*p* < 0.05), whereas no significant differences were detected among R_20_, R_40_ and CK. The pronounced enrichment of c-p2 taxa therefore suggests that intensive resource inputs and environmental modification within the mycelium-dense zone selectively favoured rapidly reproducing and disturbance-tolerant colonisers, while the increasing representation of higher-c-p taxa away from the fruiting bodies indicated progressive community recovery toward a more structured state. Taken together, the mycelium-dense zone created by understory morel cultivation functioned as a spatially concentrated resource hotspot in which fungal substrates and microbially mediated resource enrichment promoted nematode proliferation, particularly that of fungivores and c-p2 colonisers. At the same time, intensive fungal metabolism was associated with resource turnover, reduced URE activity and local acidification, potentially imposing strong environmental filtering on nematode assemblages. The coexistence of multiple trophic groups further indicates that this zone supported intensified trophic interactions, although its strong dominance by opportunistic taxa distinguished it from the more mature food-web structure observed farther from the fruiting bodies. Furthermore, the high nematode density in this zone may attract nematophagous microorganisms, potentially exerting top-down regulatory pressure on nematode populations and thereby adding another regulatory layer to the trophic dynamics of this hotspot.

### 4.2 Spatial variation in soil nematode diversity and ecological functional indices

Understory morel cultivation generated pronounced spatial differentiation in nematode diversity and ecological functional indices, which provide sensitive indicators of soil food-web organisation and ecosystem recovery (Pan et al., 2024). The Shannon–Wiener diversity index (*H*) and evenness index (*J*) were highest in R_40_, whereas the Margalef richness index (*D*) was comparatively high in both R_40_ and CK and lowest in R_20_. In contrast, the dominance index (*λ*) reached its maximum in R, revealing marked spatial heterogeneity in community organisation. The strong enrichment of opportunistic fungivores in R resulted in pronounced dominance by a limited number of taxa and, consequently, a less even community structure (Pan et al., 2024; Zheng et al., 2023). As the direct influence of the mycelium weakened with increasing distance, the dominance of these opportunistic groups declined and the relative representation of other trophic and higher-c-p groups recovered, resulting in greater community evenness and diversity (Pan et al., 2024).

RDA identified SOC and URE as major environmental correlates of nematode diversity, with community evenness positively associated with both variables. The comparatively high SOC level in R_40_ may have provided a more buffered resource environment capable of supporting a broader range of nematode life-history strategies. By contrast, intense mycelial metabolism and rapid resource turnover in R may have favoured a limited number of fast-growing opportunistic taxa, thereby reducing evenness (Vendig et al., 2023). The negative association between SOC and dominant nematode genera in the PLS-SEM model was consistent with this spatial pattern, suggesting that variation in soil carbon availability contributed to the redistribution of dominant taxa across the mycelial gradient.

Spatial variation in enzyme activity provides an additional explanation for the high community evenness observed in R_40_. URE activity was relatively high at this distance and was positively associated with nematode evenness. Enhanced urease activity can promote organic-nitrogen mineralisation and increase nitrogen availability, potentially reducing resource competition among trophic groups (Huang T. et al., 2025; Huang X. et al., 2025). Such conditions may generate a comparatively resource-rich and environmentally buffered microhabitat that supports the coexistence of multiple nematode groups rather than the numerical dominance of a small number of opportunistic taxa.

The ecological functional indices provided further support for this spatial response. Relative to CK, *SI* and *EI* were elevated in R_20_ and R_40_ and reached their highest values in R_40_, particularly in the case of *SI*. These patterns suggest that the effects of morel cultivation extended beyond the immediate mycelium-dense zone and were associated with enhanced food-web structure and resource responses at greater distances (Zhang et al., 2024). R exhibited the highest *WI* value (2.15), consistent with the strong response of microbivorous nematodes, particularly fungivores, in the mycelium-dense zone. Moreover, the comparatively low *NCR* in R indicated a greater contribution of the fungal decomposition channel relative to the more bacterially dominated pattern observed at greater distances (Jiang et al., 2018). Together, these indices support the conclusion that direct fungal resource inputs strengthened the fungal-associated trophic pathway immediately around the fruiting bodies.

In contrast, *MI* was lowest in R (2.25), indicating that the mycelium-dense zone remained subject to sustained ecological disturbance despite its high biological activity. *MI* is highly sensitive to soil disturbance, and low values generally reflect the dominance of low-c-p opportunistic taxa under disturbed or strongly resource-enriched conditions (Bongers, 1990). The low *MI* observed in R therefore corroborates the pronounced dominance of c-p2 nematodes and indicates that high resource availability did not necessarily correspond to a more mature food web. By comparison, R_40_ combined the highest *SI* and evenness with the lowest dominance, suggesting that reduced direct mycelial disturbance at this distance allowed the food web to develop toward a more structurally complex and comparatively stable state.

Beyond these spatial patterns, the decline in omnivorous-predatory nematodes in the mycelium-dense zone signals local soil faunal perturbation and reduced ecosystem buffering capacity. This community simplification may affect subsurface ecosystem functioning via three interrelated mechanisms. First, simplified nematode assemblages can weaken top-down regulation of microbial communities, leading to shifts in nutrient mineralisation processes (Yeates, 2003). Second, modifications to detritus-based energy pathways may alter carbon and nitrogen cycling efficiency (De Vries et al., 2013). Third, the loss of nematode species diversity reduces functional redundancy and may lower ecosystem resilience toward subsequent environmental disturbances (Ferris, 2010). Under large-scale cultivation, these biotic shifts may modify nutrient availability and raise the risk of continuous-cropping obstacles in subsequent cultivation cycles (Liu et al., 2022). These findings provide early-warning implications for potential ecological risks associated with large-scale *M. sextelata* cultivation.

Overall, the spatial gradient associated with understory morel cultivation strongly differentiated nematode diversity and ecological functioning. The mycelium-dense zone was characterised by resource-driven dominance of opportunistic fungivores and a simplified community structure, whereas increasing distance from the fruiting bodies was accompanied by greater representation of higher-c-p taxa and increases in community diversity and evenness. These spatial patterns were closely associated with SOC and URE and indicate that morel cultivation can simultaneously intensify resource-driven biological activity while generating contrasting effects on food-web maturity at different spatial scales. Spatial variation in nematode energy-flow structure under understory morel cultivation.

### 4.3 Spatial variation in nematode energy-flow structure under understory morel cultivation

Energy flux provides an integrative measure of multitrophic energy transfer within soil food webs and links biodiversity with ecosystem functioning, making it a sensitive indicator of soil ecological condition (Zhang et al., 2024). In the present study, understory morel cultivation substantially altered total nematode energy flux, with the greatest increase relative to CK occurring in R. Among trophic groups, bacterivores contributed the greatest energy flux and biomass, followed by fungivores and plant parasites, whereas omnivores–predators contributed the least. All trophic groups reached their maximum energy flux in R. Thus, although fungivores dominated the community numerically in the mycelium-dense zone, energy transfer was primarily channelled through bacterivores, consistent with the expectation that bacterial pathways can dominate energy flow under conditions of abundant labile resources (Nong et al., 2026).

Low-molecular-weight carbon compounds released during fungal growth, including soluble organic carbon, amino acids and organic acids, can be rapidly assimilated by opportunistic bacteria, leading to increased bacterial biomass and enhanced resource availability for bacterivorous nematodes (Barnes et al., 2014). Energy transfer through soil food webs is jointly regulated by consumer biomass, metabolic demand and trophic-transfer efficiency (Barnes et al., 2018). In this context, the greater taxonomic richness of bacterivores than fungivores in the present study—six versus two taxa, respectively (Supplementary Table S3)—is consistent with the possibility that bacterivores occupied a broader range of resource niches and collectively exploited bacterial production more efficiently. With increasing distance from the mycelium-dense zone, nematode energy flux generally declined, although R_40_ remained above CK whereas R_20_ did not. This spatial response further suggests that the magnitude of nematode-mediated energy transfer depended on the local intensity of fungal-derived resource inputs and is consistent with the identification of SOC as a major predictor of nematode energy flux in the RDA.

A notable finding was that fungivores, despite their high relative abundance, exhibited lower energy flux than bacterivores. Their biomass was also lower in R_20_ and R_40_ than in CK, whereas it increased in R. This apparent decoupling between relative abundance and energy flux highlights the importance of considering taxon-specific body size and metabolic activity rather than inferring functional importance from abundance alone. Species of *Aphelenchoides* exhibit pronounced feeding preferences among fungal taxa, and secondary metabolites produced by some fungi can exert toxic or repellent effects on nematodes (Ruess et al., 2000). The dense mycelial network in R may therefore have simultaneously provided abundant fungal food resources while exposing fungal-feeding nematodes to bioactive metabolites capable of restricting the proliferation of sensitive taxa (Yin et al., 2024). The detection of only two fungivorous taxa is consistent with, although does not by itself demonstrate, such selective effects. Consequently, fungivore energy flux increased in R but to a much smaller extent than bacterivore flux. At R_20_ and R_40_, declining mycelial resource availability, potentially combined with residual chemical effects, may have contributed to the observed reduction in fungivore biomass.

Plant parasitic nematodes represented the third-largest component of energy flux and reached their maximum in R. This response may partly reflect changes in nutrient availability and fine-root development induced by fungal activity (Vargas-Maya et al., 2022). Omnivores–predators, which occupy higher trophic positions, consistently exhibited the lowest biomass and energy flux, in accordance with the progressive loss of available energy across successive trophic transfers (Vargas-Maya et al., 2022). Their biomass declined in R_20_ and R_40_ relative to CK but increased in R. The high energy fluxes of bacterivores, fungivores and plant parasites in R may have increased prey availability and thereby strengthened the upward transfer of energy to higher trophic levels. Enhanced transfer of energy to upper trophic levels is generally associated with greater trophic connectivity within soil food webs (Huang T. et al., 2025; Huang X. et al., 2025). Nevertheless, this local increase in trophic transfer did not correspond to the highest overall food-web maturity, which was instead observed in R_40_. This distinction highlights that high rates of energy throughput and high structural maturity represent different dimensions of soil food-web functioning.

Collectively, these findings suggest that the spatial distribution of morel mycelia shaped nematode biomass and energy flow through the combined effects of resource-input gradients, bacterially mediated energy transfer and potential fungal chemical interactions. The immediate vicinity of the fruiting bodies functioned as a high-throughput energy hotspot dominated by rapid resource processing, whereas food-web organisation became more structurally mature at greater distances from the centre of mycelial activity. These results provide a more integrated understanding of spatially explicit energy-flow dynamics in soil food webs under understory morel cultivation.

Nevertheless, since this study was conducted at a single point in time, we cannot rule out the possibility of interannual fluctuations in the nematode community. Our findings primarily reflect the short-term spatial disturbance patterns caused by morel cultivation, rather than a stable, long-term ecosystem state. Further multi-site, multi-year surveys are needed to better assess the lasting ecological impacts of repeated, large-scale morel cultivation.

## 5 Conclusion

The effects of understory morel cultivation on soil nematode communities extended beyond simple changes in abundance and instead reflected strong spatial filtering associated with the distribution of fungal mycelia. The mycelium-dense zone (R) supported the greatest total nematode abundance, pronounced enrichment of fungivores and the local occurrence of several otherwise undetected genera. PCA further demonstrated a clear separation of the R community from those at greater distances, confirming a strong spatial dependence of nematode community assembly.

Total nematode energy flux and the fluxes of individual trophic groups reached their maxima in R, whereas total energy flux in R_20_ fell below that in CK, revealing pronounced spatial heterogeneity in energy transfer. Bacterivores contributed the greatest biomass and energy flux, indicating that a substantial proportion of available carbon entered the nematode food web through the bacterial channel. At the same time, the R community was overwhelmingly dominated by c-p2 colonisers (76.09%), exhibited the highest community dominance and had the lowest *MI*, indicating persistent resource-driven disturbance. With increasing distance from the fruiting bodies, the representation of higher-c-p taxa increased, and R_40_ exhibited the greatest evenness, diversity and *SI*, indicating a transition toward a more structured and comparatively stable food web.

URE and SOC emerged as the principal environmental predictors of nematode responses, individually explaining up to 49.1 and 41.9% of the variation in different components of the nematode community, respectively. The PLS-SEM further revealed a cascading pathway in which spatial distance from the mycelium-dense zone was associated with changes in SOC and enzyme activity, which in turn were linked to nematode community composition, trophic structure, energy flux and ecological functioning. These findings therefore support a conceptual pathway of “mycelial spatial gradient → soil properties → nematode community and food-web functioning”.

By incorporating nematodes into the ecological assessment of morel cultivation systems, this study extends the resource-hotspot framework to interactions between macrofungi and soil fauna and demonstrates that intensive fungal cultivation can generate sharply contrasting patterns of biological activity and food-web organisation over remarkably small spatial scales. However, this study focused solely on nematodes and did not include an analysis of soil protists. Soil protists perform important and sometimes dominant ecological functions in soil ecosystems, playing a key role particularly in promoting nutrient mineralisation, regulating microbial communities, and driving energy flow in microfood webs (Clarholm, 1981; Feest, 2006). Incorporating soil protists in future studies would further improve our understanding of the integrated soil micro-food web under morel cultivation.

Future studies combining stable-isotope tracing, such as ^13^C-labelled fungal mycelia, with molecular ecological network approaches will be important for directly resolving the transfer and allocation of fungal-derived carbon among nematode trophic groups and for elucidating the coupling between species interactions and energy transfer within the mycelium-associated micro-food web. Combined with quantitative analyses of soil protozoa, this approach would enable the development of a multi-trophic-level integrated assessment framework encompassing protozoa, nematodes, and microorganisms, thereby providing a more comprehensive understanding of the driving effects of large-scale fungal cultivation on energy flows in soil ecosystems.

## Data Availability

The raw dataset has been uploaded to Figshare, and the reserved DOI is: https://doi.org/10.6084/m9.figshare.33539272.
