# Supplementary material for: Effects of mycelial spatial gradients on soil nematode community structure and ecological functioning under understory morel cultivation
Source: Front Microbiol. 2026 Sep 30;17:1948707. doi: 10.3389/fmicb.2026.1948707 (PMC13661972; doi:10.3389/fmicb.2026.1948707)
Supplement: Supplementary file 1 [file Supplementary_file_1.DOCX]

Supplementary Material

# 1 Supplementary Tables

**Table S1. Importance ranking of explanatory power and contribution of influencing factors.**

| Influence factors |  | Explains/% | Contribution degree/% | F | P |
| --- | --- | --- | --- | --- | --- |
| Dominant genera influence factors | URE | 49.1 | 57.5 | 9.6 | 0.008 |
|  | POC | 13.5 | 15.8 | 3.2 | 0.032 |
|  | EC | 7.1 | 8.3 | 1.9 | 0.152 |
|  | ROC | 4 | 4.7 | 1.1 | 0.392 |
|  | SOC | 4.9 | 5.8 | 1.4 | 0.28 |
|  | TN | 4.4 | 5.1 | 1.3 | 0.32 |
|  | TP | 2.5 | 2.9 | 0.7 | 0.578 |
| Indices influence factors | URE | 36.6 | 48.5 | 5.8 | 0.006 |
|  | SOC | 12.8 | 17 | 2.3 | 0.06 |
|  | TP | 7.3 | 9.6 | 1.3 | 0.264 |
|  | ROC | 7.3 | 9.7 | 1.4 | 0.196 |
|  | EC | 5.2 | 6.9 | 1 | 0.376 |
|  | TN | 5.1 | 6.8 | 1 | 0.438 |
|  | POC | 1.1 | 1.5 | 0.2 | 0.928 |
| Nutritional Groups and c-p Values influence factors | SOC | 41.3 | 54.8 | 7 | 0.002 |
|  | URE | 15.7 | 20.8 | 3.3 | 0.006 |
|  | TN | 6.1 | 8.1 | 1.3 | 0.308 |
|  | TP | 3.4 | 4.4 | 0.7 | 0.544 |
|  | ROC | 3.8 | 5 | 0.8 | 0.508 |
|  | POC | 2.3 | 3.1 | 0.4 | 0.732 |
|  | EC | 2.9 | 3.8 | 0.5 | 0.636 |
| Energy flux influence factors | SOC | 41.9 | 56 | 7.2 | 0.002 |
|  | URE | 7.2 | 9.7 | 1.3 | 0.298 |
|  | POC | 9.3 | 12.4 | 1.8 | 0.166 |
|  | EC | 7.8 | 10.4 | 1.6 | 0.174 |
|  | ROC | 4.5 | 6 | 0.9 | 0.48 |
|  | TP | 2.4 | 3.2 | 0.4 | 0.688 |
|  | TN | 1.7 | 2.3 | 0.3 | 0.802 |

**Table S2. The effect fairy ring distance on soil nutrients**.

| Soil physical and chemical properties | R | R_20_ | R_40_ | CK |
| --- | --- | --- | --- | --- |
| pH | 7.226±0.020a | 7.312±0.010b | 7.371±0.023c | 7.544±0.014d |
| EC (μs·cm^-1^) | 178.000±2.309bc | 164.667±5.696a | 173.333±1.764ab | 188.000±2.309c |
| Total P (mg·g^-1^) | 1.853±0.072a | 1.723±0.052a | 1.817±0.052a | 1.917±0.048a |
| Total N (mg·g^-1^) | 4.389±0.171a | 4.417±0.208a | 4.236±0.141a | 4.031±0.048a |
| SOC (mg·g^-1^) | 46.177±4.397a | 68.557±2.291c | 57.150±0.752b | 64.080±3.882bc |
| ROC (mg·g^-1)^ | 0.617±0.018a | 0.960±0.010c | 0.610±0.015a | 0.737±0.026b |
| POC (mg·g^-1^) | 0.783±0.097 | 0.943±0.043 | 0.747±0.032 | 0.767±0.027 |
| MOC (mg·g^-1^) | 45.393±4.323a | 67.613±2.250c | 56.403±0.752b | 63.313±3.905bc |
| CAT (ml·g^-1^) | 11.527±0.077a | 12.027±0.116b | 12.293±0.096b | 12.080±0.023b |
| CBH (nmol·g^-1^·h^-1^) | 32.258±0.938c | 21.508±0.467a | 31.222±0.618bc | 29.865±0.326b |
| BG (nmol·g^-1^·h^-1^) | 61.143±0.426c | 52.220±0.438a | 59.955±0.911bc | 58.128±0.664b |
| URE (mg·d^-1^·g-1) | 0.830±0.010a | 1.17±0.015b | 1.310±0.010c | 1.153±0.019b |

R, Soil zone 0 cm from *Morchella* growth site; R_20_, Soil zone 20 cm from Morchella growth site; R_40_, Soil zone 40 cm from *Morchella* growth site. CK, Soil zone without *Morchella* cultivation. Different lowercase letters in the same column indicate significant differences among rhizosphere distances (*P*< 0.05).

**Table S3. Composition of soil nematode communities**

| Nutritional Type | Genus name | c-p value |  | Proportion/% | | |
| --- | --- | --- | --- | --- | --- | --- |
|  |  |  | R | R_20_ | R_40_ | CK |
| Plant-parasites  PP | *Dolichodorus* | 3 | 10.56 | 12.82 | 17.53 | 1.89 |
|  | *Tylenchorhynchus* | 2 | 12.11 | 7.69 | 11.34 | 14.15 |
|  | *Paratylenchus* | 2 | 0.31 | 0.00 | 0.00 | 0.00 |
|  | *Tylenchus* | 2 | 0.00 | 3.85 | 7.22 | 1.89 |
|  | *Psilenchus* | 2 | 0.00 | 0.00 | 4.12 | 0.00 |
|  | *Cephalenchus* | 3 | 7.14 | 11.54 | 6.19 | 13.21 |
|  | *Filenchus* | 3 | 0.00 | 0.00 | 2.06 | 0.94 |
|  | *Radopholus* | 3 | 0.31 | 0.00 | 0.00 | 0.00 |
| Fungivores  Fu | *Aphelenchoides* | 2 | 43.48 | 39.74 | 18.56 | 37.74 |
|  | *Aphelenchus* | 2 | 16.46 | 12.82 | 9.28 | 15.09 |
| Bacterivores  Ba | *Pseudorhabditis* | 2 | 0.00 | 0.00 | 0.00 | 0.94 |
|  | *Monhystera* | 2 | 0.31 | 0.00 | 0.00 | 0.00 |
|  | *Heterodera* | 3 | 5.90 | 11.54 | 11.34 | 8.49 |
|  | *Alaimus* | 4 | 0.00 | 0.00 | 5.15 | 0.94 |
|  | *Diplogaster* | 2 | 0.00 | 0.00 | 0.00 | 0.94 |
|  | *Rhabditis* | 1 | 0.00 | 0.00 | 6.19 | 0.00 |
| Omnivores- Predators  OP | *Odontopharynx* | 2 | 2.17 | 0.00 | 1.03 | 0.94 |
|  | *Lotonchus* | 4 | 0.93 | 0.00 | 0.00 | 2.83 |
|  | *Eudorylaimus* | 4 | 0.31 | 0.00 | 0.00 | 0.00 |

# 2 Supplementary Figure

**Fig. S1 Average daily soil temperature in the 0–10 cm soil layer during *Morchella sextelata* cultivation**
